# The role of Na_V_ channels in synaptic transmission after axotomy in a microfluidic culture platform

**DOI:** 10.1038/s41598-019-49214-w

**Published:** 2019-09-09

**Authors:** Nickolai Vysokov, Stephen B. McMahon, Ramin Raouf

**Affiliations:** Wolfson Centre for Age-Related Diseases, Institute of Psychiatry, Psychology & Neuroscience, King’s College London, London, SE1 1UL United Kingdom

**Keywords:** Lab-on-a-chip, Cellular neuroscience, Chronic pain, Ion channels in the nervous system, Synaptic transmission

## Abstract

Voltage gated sodium channels are key players in aberrant pain signaling and sensitization of nociceptors after peripheral nerve injury. The extent to which sodium channel activity after injury contributes to synaptic transmission at the first pain synapse however remains unclear. To investigate the effect of axotomy on synaptic transmission between dorsal root ganglia neurons and dorsal horn neurons, we reconstructed the first pain synapse in a novel microfluidic based compartmentalized cell culture system, which recapitulates the connectivity of peripheral pain signaling. We show that following axotomy of the distal axons, inhibition of Na_V_1.7 and Na_V_1.8 sodium channels in incoming presynaptic DRG axons is no longer sufficient to block activation of these axons and the resulting synaptic transmission to dorsal horn neurons. We found that blockade of Na_V_1.6 activity is highly effective in reducing activation of incoming axons contributing to synaptic transmission after axotomy of DRG neurons. The microfluidic culture system described here offers an *in vitro* platform to recapitulate and study the first pain synapse.

## Introduction

Perception of pain relies on transduction of nociceptive stimuli in the peripheral endings of dorsal root ganglion (DRG) neurons and transmission of these action potentials to dorsal horn (DH) neurons of the spinal cord in the central nervous system. Voltage gated sodium channels (Na_V_s) are critical for generation and propagation of action potential in sensory neurons^1^. Na_V_1.7, Na_V_1.8, and Na_V_1.9 are predominantly expressed in DRG neurons and have been the focus of drug discovery efforts due to their role nociception^2,3^ and neuropathic pain conditions^4–7^.

Mice lacking Na_V_1.8 and Na_V_1.7 channels exhibit deficits in nociception, as well as in inflammatory and neuropathic pain models^8,9^. Loss-of-function mutations of Na_V_1.7 are known to result in complete insensitivity to pain in humans^10–13^ and gain of function mutations in Na_V_1.8 and Na_V_1.7 channels may contribute to painful peripheral neuropathies^14,15^. However, drugs that effectively block these channels have so far proven ineffective in clinical trials on neuropathic pain^16^.

Although the contribution of Na_V_1.8 and Na_V_1.7 channels to excitability of sensory neurons is well established, their role in synaptic transmission remains unclear. Modulation of pain at the first pain synapse is an effective route to pain relief. Drugs such as gabapentin and opioids, which attenuate neurotransmission between DRG and DH neurons, are highly effective in the clinic^17,18^. Their side effects, desensitization and addictive nature, however, call for development of other strategies for blocking the synaptic transmission.

Studies on the synapses between nociceptive DRG neurons innervating DH neurons in the spinal cord, i.e. the first pain synapse, have been hampered by lack *in vitro* of model systems to capture the physiology of the system^19^. Current cell culture models allow tight control of the microenvironment of the synapses, and can be used to investigate how synapse formation is modulated through interactions with other neurons or non-neuronal cells^19^. However, neither localized pharmacological interventions nor axonal injury are possible with these cultures. Using a microfluidic compartmentalization technology where microfluidic channels with a very small cross-section were used to separate cell bodies from axons, was pivotal for allowing short-term and long-term manipulations of axons separately from cell bodies^20–22^. This concept was also successfully used to show that damaging DRG axons by axotomy can make them chronically more sensitive to depolarizing stimuli^23^. Thus, the ability to independently control the axonal microenvironment, apply and wash off defined concentrations of any soluble compound, and to perform distal axotomy of DRG neurons, make this microfluidic compartmentalization a uniquely versatile and robust tool for studying molecular mechanisms involved in modulation of the synaptic function in response to axonal damage.

In order to investigate the role of Na_V_s in synaptic transmission, we developed a culture model using DRG and DH neurons in a three-compartment microfluidic platform. We characterized the platform to verify the identity of the DRG neurons, their ability to send axons bilaterally and to convey a stimulus from far compartment (Periphery) to the DH neurons in the near compartment (DH compartment). We show that the DRG neurons form functional synapses with the DH neurons, recapitulating the two components of the peripheral pain pathway. We found that while blocking pre-synaptic Na_V_1.7 and Na_V_1.8 channels is effective in reducing synaptic transmission in un-injured cultures, the same blockers are ineffective in cultures where the DRG axons in the Periphery compartment had been axotomized. We have investigated further the expression of other Na_V_s to explain the lack of efficacy of Na_V_1.7 and Na_V_1.8 blockers in injured DRG neurons. We propose that the changes in pre-synaptic activity and pharmacological profile observed in this microfluidic model of first pain syanpase, should be considered when studying mechnisms of neuropathic pain.

## Results

### Reconstituting the peripheral nociceptive pathway using microfluidic co-cultures of DRG and DH neurons

**Figure 1:**
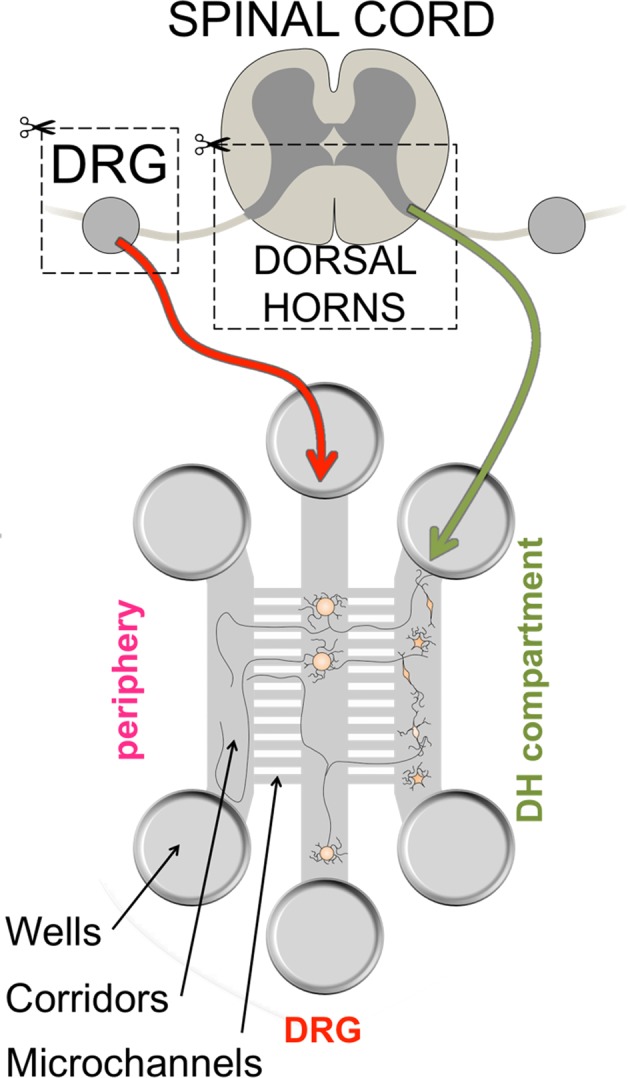
Schematic of the microfluidic co-culture setup. Embryonic (E16) rat dorsal root ganglia (DRG) and spinal cord dorsal horns (DH) were extracted, digested, dissociated and plated into different compartments of a microfluidic device in serum-free medium with NGF and AraC and allowed to mature for 12–16 DIV.

In order to investigate synaptic transmission between DRG and DH neurons after axonal injury, we established a compartmentalized co-culture model to re-capitulate the relevant aspects of peripheral nociceptive circuitry in a dish. Rat embryonic DRG and DH neurons were dissociated and cultured in separate compartments of a three-compartment microfluidic device. One compartment contained no cells (only DRG axons), dissociated DRG neurons were cultured in the middle compartment and DH neurons were grown in the third (Fig. 1). So, we refer to these compartments as Periphery, DRG and DH respectively.

**Figure 2:**
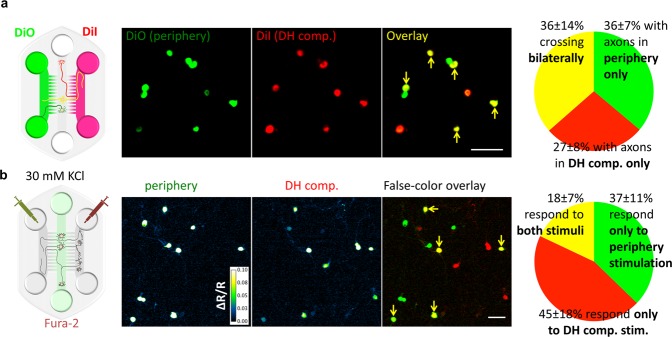
DRG neurons traverse bilaterally into Periphery and DH compartments in the microfluidic co-cultures. (**a**) To examine axonal crossing, the tracer dyes DiO (green, added to the Periphery compartment) and DiI (red, added to DH compartment) are taken up by the DRG neurons and reveal cells with neurites in both compartments in yellow (yellow arrows point to examples) as in schematic and representative image. Quantification (*n* = 4 independent cultures) reveals that 36% ± 14% (SEM) of all labeled cells can take up both dyes indicating that they have crossed bilaterally. (**b**) The DRG neurons could be activated (detected by live ratiometric Ca^2+^ imaging, where the images have been processed to highlight changes in Ca^2+^ levels relative to baseline, see “Methods” for details) by mild depolarization with 30 mM KCl applied to Periphery (left panel) or DH (middle panel). After false-coloring and overlay, cells in yellow (yellow arrows) respond to both stimuli. Quantification reveals that 18% ± 7% (SEM, *n* = 6) of DRG neurons responding to either stimulus are able to respond to stimulation of their axons from Peripheral as well as DH compartments indicating bilateral crossing of axons able to conduct action potentials. Scale bars are 100 µm.

The cultures were maintained for 12–16 days by which time many of the markers for mature DRG neurons, such as expression of TRPV1 (Figure S1), Na_V_1.8 (Figure S2), the ability to bind IB4 (Figure S1b), and the ability of a subset of DRG neurons to respond to capsaicin stimulation (Figure S1a). DRG axons traversed micro-channels from DRG compartment to both Periphery and the DH compartments (Figures S2 and S3). We traced DRG neurons with axons traversing into both fluidically isolated Periphery and DH compartments using tracer DiI and DiO. In this manner we labeled DRG soma that had axons leading into the Periphery compartment with DiO and into the DH compartment with DiI and found that 36 ± 14% of DRG neurons had crossed bilaterally into the Periphery and DH compartments (Fig. 2a).

The DRG axons in both compartments were able to conduct action potentials and activation of the DRG axons by a depolarizing stimulus (30 mM KCl) from either Periphery or DH compartments could elicit calcium transients in DRG soma compartment (Fig. 2b). We found that on average 18 ± 7% of DRG soma responding to stimulation in either Periphery or DH compartments have also responded to stimulation in the other compartment (Fig. 2b) indicating bilateral crossing of electrically competent axons from these DRG neurons. We further examined the response properties of these axons and found that past 12 DIV, a subset of these axons responded robustly to stimulation with capsaicin (Figure S4a). Hence, the DRG axons in microfluidic cultures recapitulated the bilateral innervation of the peripheral targets and the DH neurons of the spinal cord.

**Figure 3:**
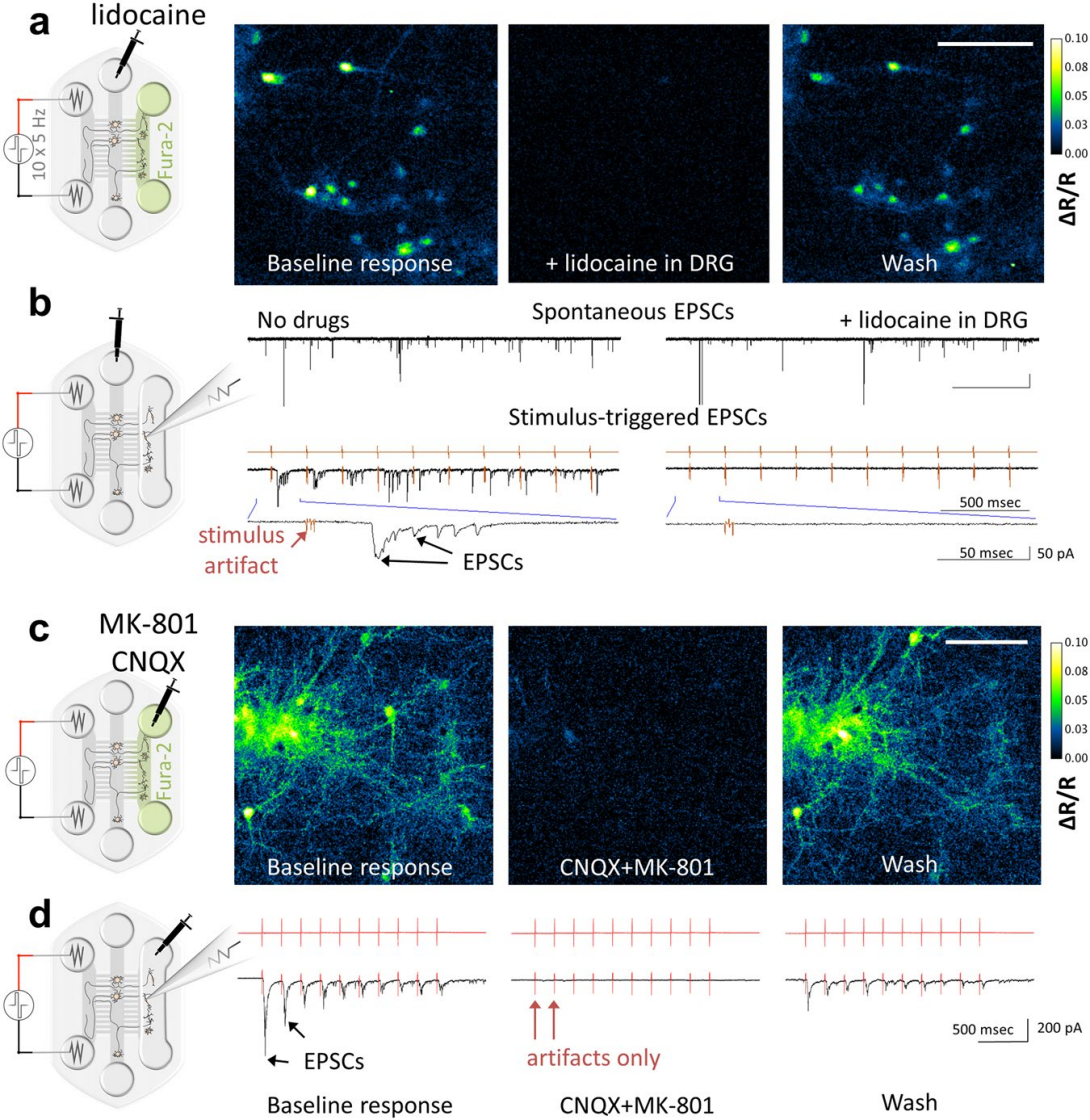
Stimulation applied to the Periphery compartment is transmitted via DRG neurons to activate DH neurons. Response of DH neurons to electrical stimulation (10 biphasic pulses at 5 Hz × 2 ms@40 µA) of Periphery was measured by calcium imaging (**a** and **c**) or by whole-cell voltage clamp recording of dorsal horn neurons (**b** and **d**). The Ca^2+^ response (average ΔR/R, see calcium imaging analysis in methods) and EPSCs are observed in a subset of DH neurons during application of the electrical stimulus (“Baseline response”). (**a** and **b**) The response in DH neurones is completely abolished after blocking signal transduction by applying 5 mM lidocaine to DRGs. Note, that lidocaine added to DRG soma did not abolish the spontaneous network activity in the DH soma compartment (**b**, “Spontaneous EPSCs”). (**c** and **d**) Blocking glutamatergic trans-synaptic transmission of signal by application of AMPA and NMDA glutamate receptor blockers (20 µM CNQX, 1 µM MK-801) to DH compartment almost completely blocks the response of the dorsal horn neurons to stimulation of the Periphery (only stimulation artifacts colored in red remain). Washing these drugs partially restores the response (right panels). Scale bars are 100 µm.

We then investigated if the DRG axons traversing to DH compartment made synaptic contacts with the DH neurons. We used electrical (10 impulses @ 5 Hz, see schematic in Fig. 3a) or chemical (30 mM KCl, Figure S4b) stimulation applied to Periphery and found that this elicited a robust transient calcium response in a subpopulation of DH neurons (Fig. 3a,c, “baseline response” and Figure S4b, top left panel) in all co-cultures tested. Electrical stimulation of the DRG axons in the Periphery also elicited excitatory post-synaptic currents in the DH neurons (EPSCs, Fig. 3c). We then used 5 mM lidocaine for blocking action potential propagation in the DRG compartment to further confirm that the responses elicited in DH neurons were due to propagation of the action potential generated in the DRG axons. We found that lidocaine completely eliminates any response of the DH neurons to the electrical stimulus (Fig. 3a,b). However, the microflouidic isolation confines lidocaine to the DRG compartment and the dorsal horn as DH neurons retained spontaneous network activity in the presence of lidocaine (Fig. 3b, top panels). The response of the DH neurons relies on glutamatergic synaptic transmission, as treatment with MK-801 and CNQX blockers of NMDA and AMPA receptors, respectively, in the DH compartment, resulted in near complete elimination of the calcium responses and the EPSCs in the DH neurons (Fig. 3c,d). These data confirm that the microfluidic co-cultures of DRG and DH neurons recapitulate many features of the peripheral pain pathway.

### The impact of axotomy on synaptic transmission to DH neurons

**Figure 4:**
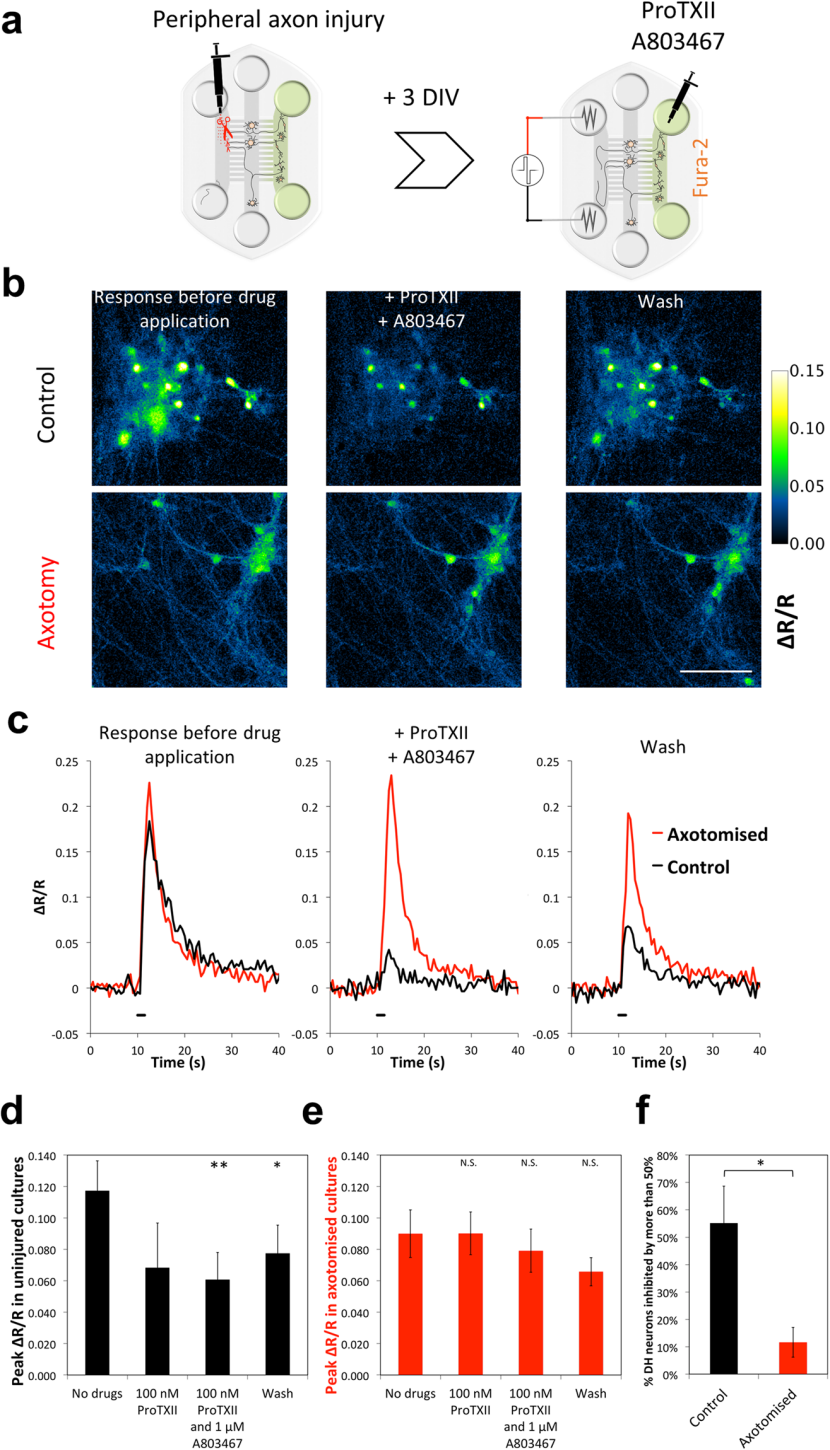
Blocking pre-synaptic Na_V_1.7 and Na_V_1.8 channels reduces response of DH neurons to stimulation of the Periphery compartment, but not in co-cultures where DRG axons had been injured. (**a**) Schematic of the experiment – DRG axons in the Periphery compartment were axotomized as described in methods and allowed to recover from acute effects of injury for 3 DIV. The response of DH neurons to electrical stimulation of Periphery with or without drugs in the DH compartment was recorded using Fura-2. (**b** and **c**) Representative images (**b**) and traces from representative DH neurons (**c**) showing responding DH neurons from control (top panel in (**b**), black trace in (**c**)) and axotomized (bottom panel in (**b**), red trace in (**c**)) co-cultures before drug application (left panels), and after application of 100 nM ProTXII (Na_V_1.7 blocker) and 1 µM A803467 (Na_V_1.8 blocker) to the DH compartment (middle panels). Note that the drugs significantly reduce or even abolish the response of some of the DH neurons in control but are ineffective in axotomized cultures. The response is partially restored after wash (right panels). (**d**–**f**) Quantification and statistical analysis of the effects of drugs on response of DH neurons to the electrical stimulus in Periphery. Peak amplitude (ΔR/R) after application of the blockers to uninjured (**d**) and injured (**e**) cultures, 3 days post axotomy, did not reduce the response amplitude of DH neurons in injured cultures. (*p < 0.05, **p < 0.01, Student’s *t*-test with Bonferroni post-hoc correction; *n* = 7 cultures) (**f**) The proportion of DH neurons that exhibited more than 50% reduction in peak amplitude (ΔR/R) after application of the blockers of ProTXII and A803467 to DH compartment was greatly reduced (*p = 0.017, Welch’s *t*-test, *n* = 7 cultures).

We set out to investigate the impact of axonal injury on Na_V_ channel activity in presynaptic axons using the microfluidic co-culture model. The microfluidic isolation of DRG axons in the Periphery compartment enabled application of shear stress^20^ to this compartment for specific injury to only those axons, which had crossed from the DRG into the Periphery compartment. This model of axonal injury *in vitro* in which distal axons were axotomized (severed) and allowed to regrow, was previously used for studying changes in Na_V_ channels in the injured axons^23^. We used the same protocol for axonal injury (see schematic in Fig. 4a). As predicted, axotomy led to increase in stress-related marker ATF3. We found that the expression of ATF3 was significantly up-regulated in the DRG soma 3 days after axotomy in the Periphery compartment, indicating the impact of axotomy on the DRG neurons after 3 days (Figures S6 and S7). The calcium responses (ΔR/R_0_) in DH neurons to stimulation of axotomized DRG axons in the Periphery compartment were comparable to uninjured co-cultures (*p* = 0.28, *n* = 7 Fig. 4b–e). This data suggests that in our model where DRG axons were axotomized and allowed to recover for 3 days, there is no significant decrease in the strength of synaptic connections between DRG and DH neurons after axotomy.

### Changes in pre-synaptic Na_V_1.7 and Na_V_1.8 pharmacology following Peripheral axotomy

We then focused on the role of Na_V_1.8 and Na_V_1.7 in synaptic transmission to DH neurons following axonal injury. We confirmed by immunocytochemistry that both channel subunits were expressed in DRG axons in all three compartments (Na_V_1.8 was expressed only in a subset of axons) but not expressed in DH neurons (Figure S3a for Na_V_1.7 and Figure S2a for Na_V_1.8). We also confirmed that Na_V_1.7 and Na_V_1.8 blockers (100 nM ProTXII and 1 µM A803467 respectively) are able to block a significant proportion of Na^+^ currents in our 12–16 DIV DRG neurons at the selected concentration (Figures S3b, c and S2b, c). We used a combination of Na_V_1.7 and Na_V_1.8 blockers in the DH compartment to examine the response of DH neurons to electrical stimulation of DRG axons in Periphery (see schematic in Fig. 4a). In uninjured co-cultures the response of DH neurons in presence of these blockers was significantly reduced on average (Fig. 4d, *p* = 0.0044, *n* = 7) and almost completely eliminated in some cells as shown in representative images and traces (Fig. 4b, top panel and 4c). Washing the blockers partially restored the response to the original signal amplitude (Fig. 4b–d, right panels and right bar). However, in axotomized cultures, application of the same inhibitors to block Na_V_1.7 and Na_V_1.8 in the DH compartment had no effect (Fig. 4b, bottom panel and 4c, red trace and 4d, *p* = 0.376, *n* = 6). By looking at the proportion of cells affected by Na_V_1.7 and Na_V_1.8 blockers we can directly observe the striking difference between the pharmacological profile of injured and uninjured cultures (Fig. 4f, *p* = 0.017, *n* = 7). These data show that synaptic transmission at the DRG-DH synapse following axotomy of distal DRG axons, can take place independently of Na_V_1.7 and Na_V_1.8 channels, and the unexpected difference, suggests aberrant Na_V_ channel activity in axotomized neurons.

### Na_V_ 1.6 is the dominant sodium channel subunit following injury to distal axons

**Figure 5:**
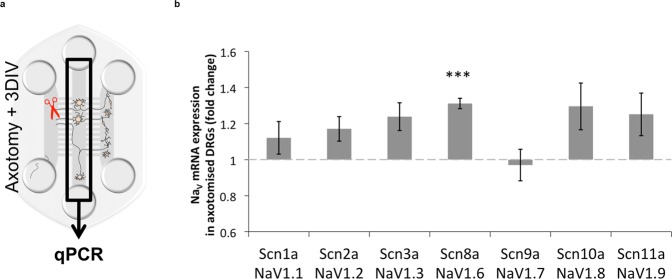
Changes in Na_V_ mRNA expression in axotomized DRG neurons. (**a**) Schematic of the experiment. 3 DIV after axotomy the RNA from axotomized and control DRG neurons was collected, and differences in expression of VGSCs were analyzed by qPCR (see methods for details). (**b**) Quantification of changes in Na_V_ mRNA expression (Na_V_1.4 and Na_V_1.5 were not expressed in both conditions) in axotomised cultures, compared to control. Na_V_1.6 (*Scn8a*) is significantly upregulated in DRG neurons following injury (*p < 0.05, One-sample *t*-test with Bonferroni post-hoc correction; *n* = 7; error bars represent SEM). See Figure S7 for relative expression levels of other genes.

We hypothesized that the observed lack of effect of Na_V_1.7 and Na_V_1.8 blockers in injured cultures could be due to changes in expression of one or more voltage-gated sodium channel subunits. The compartmentalization allowed for extraction of RNA material specifically from the DRG compartment to screen for changes at the mRNA level in the DRGs following axotomy (see Methods and Fig. 5a).

Since Na_V_1.4 and Na_V_1.5 are not expressed by DRG neurons (also confirmed by PCR, Figure S7A), we focused our analysis on Na_V_1.1–1.3 and 1.6–1.9. The most highly expressed VGSC was Na_V_1.7 (*Scn9a*, Figure S7A which is expressed by the majority of DRG neurons (also see immunocytochemistry in Figure S3A) and it was not significantly different from the control. While the expression of *Scn10a*, second highest expressed channel in our analysis), *Scn11a*, *Scn2a*, and *Scn3a* followed a trend of being up-regulated, they did not pass statistical significance following adjustment for multiple comparisons (*p* < 0.10). It is possible that higher changes in levels of expression were masked by DRG cells that did not cross into the Periphery compartment (~60 ± 8% of all DRGs) and it was not possible to separate these out. However, interestingly, despite this disadvantage, the expression of Na_V_1.6 (*Scn8a*) was found to be significantly upregulated by 25% in DRG cells (Fig. 5b, *p* < 0.05, *n* = 7, *t*-test with Bonferroni post-hoc correction). We confirmed expression of Na_V_1.6 in DRG soma, axons in the Periphery and DH compartments by immunocytochemistry and we also found DH neurons to strongly express Na_V_1.6 (Figure S5a). These data suggested that the upregulation of Na_V_1.6 in presynaptic axons following injury to distal axons could contribute to synaptic transmission to DH neurons.

### Blocking Na_V_1.6 is effective in reducing activation of DRG axons that “innervate” DH compartment

**Figure 6:**
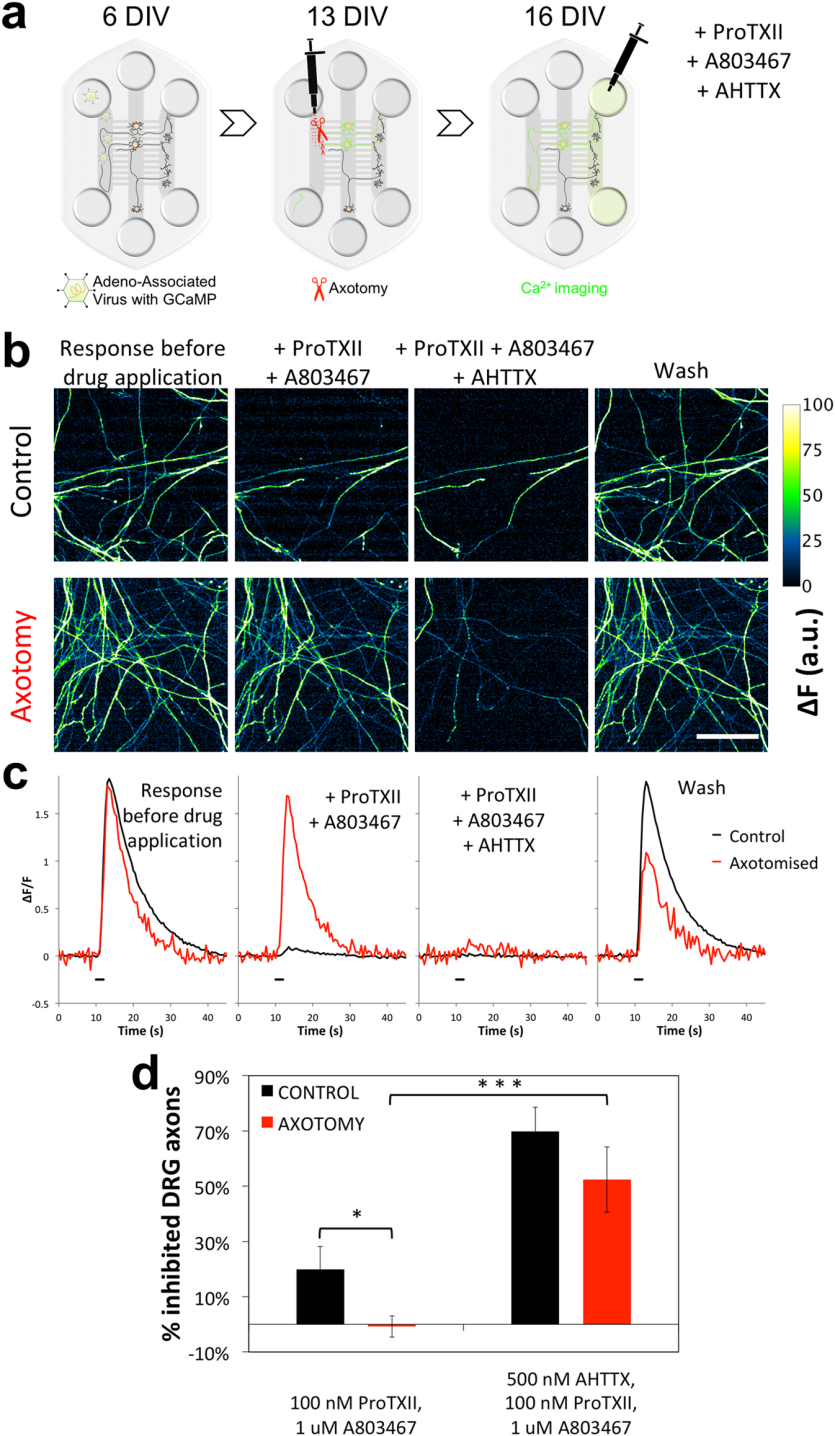
4,9-AHTTX is effective at blocking Ca^2+^ responses in DRG axons, which are not blocked by ProTXII and A803467. (**a**) Schematic of the protocol. AAV particles encoding for GCaMP6s gene were added to Periphery compartment and taken up by DRGs with axons that have crossed by 6 DIV. At 12–13 DIV the axons were injured (as above). The effect of peripheral injury on the DRG axons in the DH compartment and their pharmacology was investigated by imaging GCaMP fluorescence response (ΔF) to electrical stimulation (10 pulses at 5 Hz × 2 ms@40 µA, biphasic). (**b**) DRG axons in DH compartment from control (top panel) or axotomized cells (bottom panel) express GCaMP and respond to electrical pulses applied to Periphery with transient increases in fluorescence (first column – “response before drug application”). Note how populations of axons disappear when treated with drugs (100 nM ProTXII, 1 µM A803467, 500 nM AHTTX), while other populations remain unaffected. (**c**) Traces showing transient Ca^2+^ spikes (expressed in arbitrary units from ΔF/F) in representative DRG axons from control (black) and axotomized (red) cultures following electrical stimulation (black bar) in presence of the same drug combinations. (**d**) Quantification of axons responding to electrical stimuli (see Methods for details of analysis). Note that, as in Fig. 4, ProTXII and A803467 are effective at blocking response in some axons in control cultures (black) in comparison to axotomized cultures (red) (*p = 0.039; Welch’s *t*-test; *n* = 9 independent experiments). Moreover, combining these blockers with AHTTX is highly effective at blocking Ca^2+^ transients in axons from axotomized cells (***p = 0.00068; Paired t-test, *n* = 9). Scale bars are 100 µm.

We next hypothesized that the changes in Na_V_ channels expression observed in the DRG neurons could underlie axotomy-induced changes in pharmacology of signal transmission to DH neurons. In order to investigate contribution of Na_V_ channels to the propagation of action potential in DRG axons innervating the DH compartment, we took advantage of a replication-deficient adeno-associated virus with the gene to encode Ca^2+^ indicator GCaMP6s in host cells. When added to the Periphery it is taken up by crossing DRG axons and the GCaMP becomes expressed throughout the whole neuron. Thus, all GCaMP positive axons in the DH compartment could only have come from bilaterally crossing DRG neurons transduced by the virus and, moreover, these would have also all been injured when axotomy was performed as the virus was added prior to the procedure (see schematic in Fig. 6a). In this way we were able to reliably identify and restrict analysis to axons from DRG neurons, which have undergone axotomy.

Electrical stimulation of DRG axons in the Periphery compartment elicited a Ca^2+^ transient response in the axons of bilaterally crossing DRGs in the DH compartment (Fig. 6b,c, left panels). These axons responded to application of KCl to the Periphery compartment. A subset of the axons responded to stimulation with capsaicin, suggesting that the major DRG neuronal subtypes have bilaterally crossing axons (Figure S4a). In this paradigm, addition of Na_V_1.7 and Na_V_1.8 blockers to the DH compartment resulted in significant reduction of the response in a subset of the DRG axons in the DH compartment (Fig. 6b,c). In axotomized cultures, however, the combination of ProTXII and A803467 had no significant effect on axonal responses (Fig. 6b,c), which is consistent with the effects observed postsynaptically on the responses of DH neurons following axotomy (Fig. 4b,c). Quantification of this pharmacological phenotype revealed a statistically significant difference between changes in number of responding axons after incubation with the two inhibitors in axotomized neurons (no effect: −1 ± 4%) and in control (reduction by 20 ± 8%; *p* = 0.039).

We then tested whether Na_V_1.6 could be mediating transmission of signals in DRG axons in the DH compartment. We used a metabolite of TTX – 4,9-Anhydrotetrodotoxin (AHTTX), which specifically blocks Na_V_1.6^24^. We found that 500 nM AHTTX partially blocks Na^+^ currents in DRG neurons (Figure S5a). We also found that the addition of AHTTX together with ProTXII and A803467 significantly reduced or completely abolished the response of a 52 ± 12% axons, which did not respond to addition ProTXII and A803467 alone in axotomized cultures (*p* = 0.002, *n* = 9). The Ca^2+^ response in the DRG axons is partially or completely restored after washing the blockers, suggesting that the reduction observed is specifically due to the addition of the combination of blockers. These data confirmed a novel role for Na_V_1.6 subunit as the dominant sodium channel, in the excitability of DRG axons innervating DH neurons following axotomy at the distal axons in a microfluidic culture model.

## Discussion

In this study we developed a novel microfluidic based culture model to investigate responses to axonal injury and found unexpected changes in Na_V_ channel activity following injury. The *in vitro* model of compartmentalized DRG and DH neuron co-culture for studying signal transmission between the peripheral and central neurons developed for this paper recapitulates the bilateral nature of DRG neurons synapsing onto the dorsal horn cells in the DH compartment and extending their axons into the Periphery compartment at the same time (Figs 1–3). Moreover, it was combined with the previously published approach for inducing chronic sensitization by injury in DRG axons^23^. The present approach has numerous advantages over other *in vitro* and *ex vivo* models studying DRG-DH communication^19,25,26^ including specific and controlled manipulations of DRG axons in the Periphery (e.g. axotomy in Figs 4–6 and viral transduction in Fig. 6) or synapses and cells in the DH compartment (pharmacology in Figs 3, 4, 6). Reconstruction of neuronal circuitry in microfluidic platform affords direct local application of pharmacological modulators with concomitant measurements of neuronal activity using fluorometry or patch clamp electrorphysiology^23^. Performing *in vivo* patch clamp electrophysiology on the spinal cord dorsal horn neurons is technically limited and suffers low throughput data collection. Moreover, the microfluidic cultures offer a platform whereby to study neuronal and synaptic responses to axonal manupilations, but also the interaction with other systems such the immune cells^23,27,28^.

In our model the cells were extracted from rat embryonic E16 neurons, which dramatically improves their long-term survival and makes the model more robust than short-term cultures from adult rat tissue. The expression of Na_V_1.7 and Na_V_1.8 channels is initially low^29^, however, over the culture period (12–16 DIV) and in the presence of NGF these cells mature, expanding in size, expressing markers for mature nociceptors with the smaller neurons binding IB4 (Figure S1b), expressing Na_V_1.7 and Na_V_1.8 subunits (Figures S3 and S4), and functional TRPV1 receptors (Figures S1 and S2). Moreover, they made functional glutamatergic synapses with the DH neurons (Fig. 3c,d) and undergo molecular changes in response to axonal injury (a corollary of peripheral nerve injury) (Figs 4–6, Figure S6). However, a caveat for using primary embryonic neurons matured in culture is that the stage of maturation achieved in culture may not directly correspond to mature neurons *in vivo*. Extension of this platform to host more mature tissue, such as postnatal neurons, would be of interest in this regard.

We showed here that a microfluidic culture platform can be used to study the modulation of synaptic transmission between DRG neurons and DH neurons. We used this platform to investigate the impact of axotomy on synaptic transmission between DRG and DH neurons. Neuropathic pain is highly prevalent in patients with peripheral nerve injuries that involve axotomy of a subset of the sensory neurons^30^ and many studies support the involvement of the axotomized subset of DRG neurons in the Spinal Nerve Ligation, Spared Nerve Injury and Chronic Constriction Injury models of neuropathic pain^30–32^. As such understanding the axotomy-induced changes in synaptic transmission is highly relevant to understanding pathological pain. Recapitulating other types of injury such as partial nerve damage, however, would be technically prohibitive in the microfluidic culture platform in its current form.

We further showed that this microfluidic platform can recapitulate the salient features of the peripheral pain pathway and hence, could be used for studying the pharmacology of the peripheral nerve injury. Furthermore, there is scope for modifying the device design for high-throughput applications in industrial screening against synaptic transmission at the first pain synapse. Thus, the microfluidic platform we describe here, is to our knowledge, the first to exploit the versatility of microfluidic devices to model injury induced functional changes in neuronal circuits *in vitro*. One important disadvantage of the models is that it does not capture the entire complexity of *in vivo* pain signaling, and the findings should ultimately be validated using animal models. Despite this caveat, the microfluidic culture platform described here goes a long way towards a physiologically relevant surrogate cell culture model to study mechanisms of pathological pain. As a surrogate for *in vivo* pain models, application of this microfluidic based *in vitro* model of neuropathic pain in the study of pain transmission after peripheral nerve damage, can ultimately lead to a significant reduction in the number of animals needed for such projects.

Our aim here was to illustrate the utility of the microfluidic model in investigating modulation of synaptic transmission between DRG and DH neurons in the DH compartment. We had reported previously that axotomizing DRG neurons in microfluidic devices lead to robust changes in DRG axons excitability at the site of damage in the periphery compartment^23^. Using our microfluidic model of peripheral pain pathway, we examined the role of Na_V_1.7 and Na_V_1.8 in trans-synaptic transmission of pain^33,34^. We showed that while specific blockers for Na_V_1.7 and Na_V_1.8 were able to effectively block signal transmission in uninjured cultures, we found that they are no longer effective in injured cultures (Fig. 4). The blockers were used at concentrations reported to optimally block their target channels with minimal off-target effect. Although, it is possible that at higher concentrations these blockers would inhibit synaptic transmission after axotomy, the potential for off-target effects limits their utility. Nevertheless, these results clearly demonstrated a significant reduction in the contribution of Na_V_1.8 and Na_V_1.7 channels to synaptic transmission following axotomy. Interestingly, Na_V_1.7 and Na_V_1.8 conditional knock-out in mice does not affect neuropathic pain in SNL model^8^. Although Na_V_1.7 loss-of-function mutations were known to completely abolish any pain signaling in uninjured patients, one case study reported injury-induced neuropathic pain in a patient with Na_V_1.7 congenital insensitivity to pain^35^. Hyperactivation of the endogenous opioid system has been suggested to underlie insensitivity to pain in patients with Na_V_1.7 null mutations^11^ and hence lack of efficacy of peripherally acting Na_V_1.7 blockers in the clinic^16^. Future experiments using more intact preparations, such as spinal cord slices from neuropathic animals, could determine whether similar changes in Na_V_ channel contribution to synaptic transmission at the first pain synapse are present in neuropathic pain conditions.

Changes in Na_V_ channel expression after injury have been reported in animal models of pain^5,7^. We explored the possibility that the observed switch away from Na_V_1.7 and Na_V_1.8 sodium channels in the microfluidic model is due to changes in Na_V_ channel expression. We found a modest trend for increase in the expression of Na_V_1.2, Na_V_1.3, Na_V_1.6, Na_V_1.8 and Na_V_1.9, however, only up-regulation of Na_V_1.6 mRNA was statistically significant. We observed that action potential-evoked calcium transients in DRG axons from axotomized DRG neurons in the DH compartment were successfully blocked when Na_V_1.6-specific blocker were used in combination with Na_v_1.7 and Na_v_1.8 blockers. The involvement of Na_V_1.6 is less well studied in neuropathic pain, but recent reports suggest a role in certain neuropathic pain models. In humans, a gain-of-function mutation of Na_V_1.6, which lowers its threshold of activation, was recently found in a case of trigeminal neuralgia^36^. As well, siRNA against Na_V_1.6 reduces mechanical hypersensitivity and allodynia in SNL and modified CCI models of neuropathic pain^37^. Chen *et al*. have reported that a significant effect of local knock-down of Na_V_1.6 on mechanical sensitivity in neuropathic pain following SNI was observed, while conditional knockout of Na_V_1.6 in Na_V_1.8 expressing neurons was partially effective^38^. Interestingly, no significant upregulation of Na_V_1.6 protein levels in DRG cell bodies was observed^37^. Our experiments show a clear change in the role of this channel in synaptic transmission after axotomy in a microfluidic model of first pain synapse. This dramatic change in the involvement of Na_V_1.6 could be result of trafficking of the channel from the cytosol to the membrane via palmitoylation of δ-catenin shown for *in vivo* injury- and chemotherapy-induced neuropathic pain models or the regulation of Na_V_1.6 activity through upregulation of Na_V_β4 auxiliary subunit in inflammatory pain^39^. Taken together, and assuming positive validation, the results here would suggest that, in cases of neuropathic pain where Na_V_1.7 and Na_V_1.8 blockers administered in conjunction with blockers of Na_V_1.6 could be highly effective in reducing pain phenotype.

This would make Na_V_1.6 an attractive potential therapeutic target for treating respective heritable cases of chronic pain and some cases of injury-induced neuropathic pain. As Na_V_1.6 is also expressed by spinal cord neurons^38^ (Fig. 6), a potential strategy would be local gene therapy^37,38,40^. Although gene therapy alone may not be sufficient for blocking signal transmission, the remaining signaling due to Na_V_1.7 and Na_V_1.8 could be blocked with existing pharmacological compounds^13,41^. Nonetheless, any therapeutic strategies should consider potential changes in mechanisms of nociceptive signaling following injury to nociceptive neurons.

In summary, this study developed a novel microfluidic based platform which captures the connectivity of the peripheral pain pathway. Using this cell culture model, we unmasked a profound shift in Na_v_ channels contributing to synaptic transmission between DRG and DH neurons, from Na_v_1.7 and Na_v_1.8 to Na_v_1.6, following severance and recovery of distal axons.

## Methods

### Materials

All salts and small molecules were purchased from Sigma, unless specified otherwise. Culture reagents were purchased from Thermo Fisher Scientific, unless specified otherwise. ProTXII was purchased from SmarTox (07PTX002) and used on the day of reconstitution; A803467 (2967) and AHTTX (6159) were purchased from Tocris and lidocaine (L5647) and capsaicin were purchased from Sigma.

### Cell culture and axotomy

The microfluidic devices (Xona Microfluidics, TCND1000) were assembled by non-plasma bonding method^42^ onto glass bottom dishes (Willco) pre-coated with 0.5 mg/ml poly-L-Lysine (P1274, Sigma) in borate buffer. After assembly the surface was coated with 40 µg/ml laminin (L2020, Sigma) in Neurobasal medium (Thermo Fisher Scientific).

Pregnant Wistar rats (Charles River) were sacrificed and E16 embryos were removed and sacrificed by decapitation. All animals were maintained in a designated facility in strict accordance to the UK Home Office Code of Practice for the Housing and Care of Animals Used in Scientific Procedures. Animals were sacrificed and tissue was collected in strict accordance to the UK Home Office regulations and procedures under Schedule 1 of Animals (Scientific Procedures) Act 1986. All procedures were approved by the Animal Welfare and Ethical Review Body at King’s College London (PPL U136).

The spinal cord was dissected from the embryos and the dorsal root ganglia (DRGs) were collected. The dorsal horn segment of the spinal cord was dissected from the lengths of the cord (the meninges were carefully removed) and placed in HBSSc (HBSS + 1% Glucose + 20 mM HEPES) +2% BSA and pooled together from different embryos. DRGs were digested in 0.125 mg/ml collagenase (C7657, Sigma) and 10 mg/ml dispase (17105-041, Thermo Fisher Scientific) in HBSSc for 15–20 minutes, while DH pieces were digested in 30 U/ml papain (P4762, Sigma) in Hibernate buffer without Ca^2+^ (BrainBits UK) for 7–10 minutes. Cells were dissociated in dissociation buffer containing 0.5 mg/ml DNAse (10104159001, Roche), 1% BSA in NB/B27 (Thermo Fisher Scientific), with fire-polished, autoclaved glass Pasteur pipettes and the debris were removed by centrifuging DRGs on 15% BSA in HBSSc or DH cells on 5% BSA in NB/B27. After re-suspension of the pellet the cells were counted and plated at 10,000 DRGs per µl and 20,000 DH neurons per µl (5 µl total) into respective compartments as shown in Fig. 1 into the microfluidic devices. Culture medium NB/B27 + GlutaMax (Life Technologies) + 50 ng/ml NGF (50385-MNAC, Life Technologies) was added after 1 hour incubation so as to minimize cell detachment. At 2 days *in vitro* (DIV) the medium was replaced completely and supplemented with 1 µM cytosine β-D-arabinofuranoside (AraC); after that the medium was replaced every 3–4 days. AraC was included to slow down the growth of non-neuronal cells in the cultures. Slight positive pressure from DRG soma compartment (by adding 40 µl more medium than in the other two compartments) facilitated bilateral spreading of the axons from the DRG cells.

Axotomy was performed as described in^20^ and Tsantoulas, *et al*.^23^ with modifications. The axons were cut at 12–13 DIV by triturating ~200 µl of NB/B27 next to both entrances of the corridor of Periphery compartment and washed. Damage of all axons was verified under a microscope and if it was incomplete, the procedure was repeated. At the end of the axotomy, both axotomized cells and control cells (medium was changed, but no trituration was applied) had medium replaced with fresh medium and placed back into the incubator for 3 days, at which point the cells were probed as described below.

### Dye tracing and viral infection

For tracing DRG axons, 1:200 DiO or DiI dyes (Thermo Fisher Scientific) dissolved in culture medium were added to the Periphery or DH compartments for 1 hour, washed and allowed to diffuse within the cells for at least 1 day before live imaging DRG neurons.

For infecting DRG cells, which have crossed into the Periphery, at 6 DIV the virus with gene encoding for calcium indicator AAV9.CAG.GCaMP6s.WRPE.SV40 (Penn Vector Core, University of Pennsylvania, Philadelphia, PA) diluted in medium to ~1.1 × 10^8^ gene copies/µl was added to the corridor under negative pressure and washed after 3DIV. Thus, any DRG axons in DH compartment, which expressed GCaMP are bilaterally crossing and moreover, where the culture was axotomized at as described above, any green axons would have come from injured cells.

### Calcium imaging

Cells cultured in microfluidic devices as described above were washed with ACSF (130 mM NaCl, 10 mM Na-HEPES, 5 mM KCl, 2 mM CaCl_2_, 1 mM MgCl_2_, 30 mM Glucose, pH 7.4; supplemented with 1 mM probenecid for imaging Fura-2). For ratiometric calcium imaging, DRG or DH neurons were loaded with 2.5 µM Fura-2 dissolved in ACSF for 1 hour in the incubator with occasional rocking and washed before imaging.

An inverted fluorescent microscope (Nikon Eclipse TE200) with 20X Plan Fluor, 0.5 NA objective was used for all live imaging experiments. For imaging GCaMP, a FITC filter was used and cells were excited at 488 nm wavelength using a Random Access Monochromator (PTI) with Xenon Short Arc 75 W lamp, while for imaging Fura-2 at 340 and 380 nm wavelengths, in both cases fluorescence images were acquired by Hamamatsu Orca FLASH 4.0 at 2 frames per second using EasyRatioPro (PTI) software. To deliver electrical stimuli, a Neurolog NL800A stimulus isolators driven by Neurolog signal generators (Digitimer) configured to deliver biphasic stimuli was connected to coiled Pt wires submerged into each wells on the side of the corridor in the Periphery compartment. It was empirically determined (data not shown) that 5 Hz biphasic pulses (2 ms for each phase) are optimal for exciting the majority of the DRG axons and a train of 10 pulses at 2x threshold amplitude (i.e. 40–80 μA) elicit a robust response in the DH neurons (concentrations of DMSO up to 0.2% were tested as a control over an imaging period of up to 3 hours). The train of pulses was delivered three times for each drug combination with at least 2.5 minutes in between the trains and at least 10 minutes after each drug application or wash sequence.

Image analysis was done in FIJI. To generate an image of responder cells, an average image of 10 frames after the stimulus was divided (or subtracted) by an average of 10 frames before the stimulus to generate ΔR/R_0_ (or ΔF) images (macro used for the analysis are deposited at https://github.com/RaoufLab/NV-ImageJ-Scripts), where ΔR/R_0_ is the change in the fluorescence intensity ratio, F_340_/F_380_ for Fura-2 experiments (or ΔF/F for GCaMP)_._ For ease of visualization “Green Fire Blue” LUT is used throughout the paper with the calibration bar next to each image. For DH neurons, cells that responded to electrical stimulation of the periphery compartment in the absence of any blockers were selected for further analysis. The mean change in ΔR/R_0_ for each ROI was then calculated for each cell in the presence of the blockers. If the ΔR/R_0_ was reduced by more than 50%, the cell was considered as responding to the blockers. Cells in which the baseline had significantly changed during the experiment were discarded. For DRG axons, a threshold mask was applied to the ratioed images of axons during electrical stimulus with no blockers, and the same threshold mask was then applied to the images from the same field responding to electrical stimulus in the presence of the blockers. The area of responding axons (where all pixels with intensity great than 50 a.u.) was compared and quantified to assess proportion of axons that were inhibited by the blockers.

### Patch clamp electrophysiology

All electrophysiology experiments were performed on an inverted Nikon Diaphot-TMD microscope with attached Scientifica CV203BU Headstage. Signal was amplified with Axoclamp 200B amplifier and passed through 5 kHz filter, before being digitized with Digidata 1320 A at 10 kHz and recorded by pCLAMP 9.2 software (all from Molecular Devices). Series resistance was monitored between incubations and recordings were discarded if exceeded 12 MOhm changed significantly or.

For measuring Na^+^ currents in DRG neurons recording electrodes were made to 2–3 MOhm and filled with K^+^-free solution containing (in mM) 110 CsCl_2_, 10 HEPES, 10 EGTA, 25 NaOH, 1 MgCl_2_, 0.1 CaCl_2_, 2 ATP-Mg (pH7.4, 250 mOsm). Neurons were perfused with bath solution containing (in mM) 130 NaCl, 3 KCl, 10 Na-HEPES, 2 CaCl_2_, 2 MgCl_2_, 10 Glucose, 5 HCl pH 7.4 (255 mOsm) with drugs as noted in the captions. The voltage-clamp protocol involved a negative step to −100 mV and depolarization to −20 mV (for experiments on Na_V_1.7 and Na_V_1.6 currents) or 0 mV (for experiments on Na_V_1.8 currents). Difference between peak current at 0 mV step and baseline current at −100 mV was used for statistical analysis.

For recording post-synaptic currents, the microfluidic devices were modified as described in^23^ to allow access of patch pipettes to DH neurons in the DH compartment. The neurons were patched in pACSF (in mM, 130 NaCl, 10 Na-HEPES, 2.5 KCl, 2 CaCl_2_, 1 MgCl_2_, 10 glucose, 5.8 HCl pH 7.4, 295 mOsm) and recording electrodes (3–5 MOhm) were filled with physiologically relevant intracellular solution (in mM, 130 K gluconate, 10 NaCl, 1 MgCl_2_, 0.2 EGTA, 10 HEPES, 1 ATP-Mg, pH 7.4 with KOH, 270 mOsm). Biphasic electrical stimuli were delivered to DRG axons in Periphery compartment with the same equipment and in the same manner as above for calcium imaging, voltage measurement was performed for first stimulation, after which the probes were disconnected to avoid interference. DH neurons that did not respond to electrical stimuli applied to Periphery were discarded, only one incubation with the drugs was performed per device.

### Immunocytochemistry

After calcium imaging described above cells were fixed in 4% paraformaldehyde in phosphate buffer for 10 minutes, washed and stored at 4 °C. The cells were blocked and permeabilized in devices in blocking buffer (5% goat serum, 5% donkey serum + 0.1% Triton-X100) for 1 hour at RT with gentle rocking prior to staining. The primary antibodies (or IB_4_-Alexa 488, Invitrogen, I21411 used at 50 µg/ml): chicken anti-GFP (Abcam, ab13970 used 1:1,000), rabbit anti-ATF3 (Novus Biologicals, NBP1-85816 used 1:200), rabbit anti-Na_V_1.6 (Alomone, ASC-009 used 1:200), mouse anti-Na_V_1.7 (Neuromab, N68/6, used 1:500), mouse anti-Na_V_1.8 (Neuromab, N134/12, used 1:200), mouse anti-beta3 tubulin (R&D Systems, MAB1195 used 1:1,000), goat anti-TRPV1 (Santa Cruz, SC-12498, used 1:500) were incubated with the cells overnight on a rocker at 4 °C. Respective secondary antibodies (all from Invitrogen and all used at 1:1,000): goat anti-chicken Alexa 488 (A11039), donkey anti-mouse Alexa 488 (A21202), donkey anti-mouse Alexa 594 (A21203), donkey anti-rabbit Alexa 594 (A21207), donkey anti-goat Alexa 594 (A11058) for 1 hr at RT on a rocker. The cells were imaged on Zeiss Imager.Z1 with 20×/0.5NA EC-Plan-NEOFLUAR objective and respective standard DAPI, FITC and TRITC filters (to allow imaging on upright microscope the devices were turned upside down) within 2–4 hours after washing.

### qPCR

DRG and DH neurons were cultured and axotomized as above, washed with PBS and all cells in DRG compartment were lysed in 20 µl volume of lysis buffer from RealTime ready Cell Lysis Kit (06366821001, Roche), diluted 1:5 and reverse transcribed using Transcriptor Universal cDNA Master (05893151001, Roche). The cDNA was used to make 1 ml of Master Mix (using LightCycler 480 Probes Master, 04707494001, Roche) distributed among 48 wells of RealTime Ready Custom Panels with optimized primers and probes for a wide range of ion channels (05532914001, configuration 100036039, Roche). The cDNA was amplified and product measured using LightCycler 480 system (Roche). The data was normalized to geometric mean of four reference genes (Actb, Gapdh, S16r and G6pd) and analyzed using conventional 2^−ΔCp^ for relative expression in control or 2^−ΔΔCp^ method for measuring fold-change after axotomy^43^.

### Statistical analysis

Paired *t*-test (Excel) was used to compare the peak response (ΔR/R, ΔF/F or I_Na_) of the cells before and after drug applications. Welch’s test (Excel) was used to compare axotomy and control peak responses (ΔR/R, ΔF/F), and protein expression using quantification of immunofluorescence.

The changes in relative expression of voltage gated sodium channels were statistically tested by one-sample *t*-test with the *p* values adjusted to account for multiple comparisons using Bonferroni post-hoc correction.

## Supplementary information

Supplementary Information

## Data Availability

The raw data, primer and probe sequences as well as live calcium imaging supporting the findings are available from the corresponding author, R.R., upon reasonable request. The code used for ICC quantification and processing of calcium imaging data is available from https://github.com/RaoufLab/NV-ImageJ-Scripts, please cite this article when using the code.
