# Supplementary material for: The role of NaV channels in synaptic transmission after axotomy in a microfluidic culture platform
Source: Sci Rep. 2019 Sep 9;9:12915. doi: 10.1038/s41598-019-49214-w (PMC6733904; doi:10.1038/s41598-019-49214-w)
Supplement: Supplementary file 1 — Supplementary Information [file 41598_2019_49214_MOESM1_ESM.pdf]

The role of Nav channels in synaptic transmission after axotomy in a microfluidic culture platform

Nickolai Vysokov[1], Stephen B McMahon[1], Ramin Raouf[1, *]

[1] – Wolfson Centre for Age-Related Diseases, King's College London, London, SE1 1UL, United Kingdom

*– Correspondence: Ramin Raouf ramin.raouf@kcl.ac.uk

Supplementary Figures

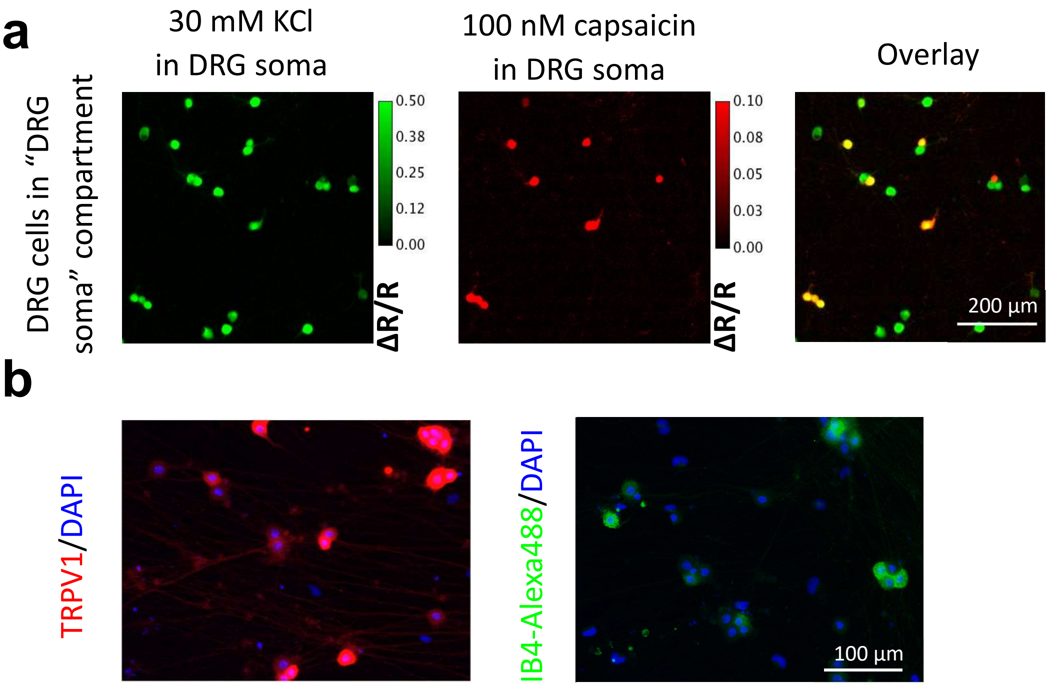

Figure S1. Characterization of DRG neurons in microfluidic cultures. (A) E16 DRG neurons co-cultured with DH neurons in compartmentalized device for 12-16 DIV possess properties of mature DRG neurons. All DRG cells loaded with ratiometric calcium indicator that respond to 30 mM KCl depolarization are colored in green (left panel), a subset of these cells colored in red (middle panel, yellow in right panel) also responded to 100 nM capsaicin. **(B)** TRPV1 expression in a subset of DRG neurons was also shown directly by immunolabelling (left panel, red color) and a small proportion of the DRG neurons bind IB4 (right panel, green color).

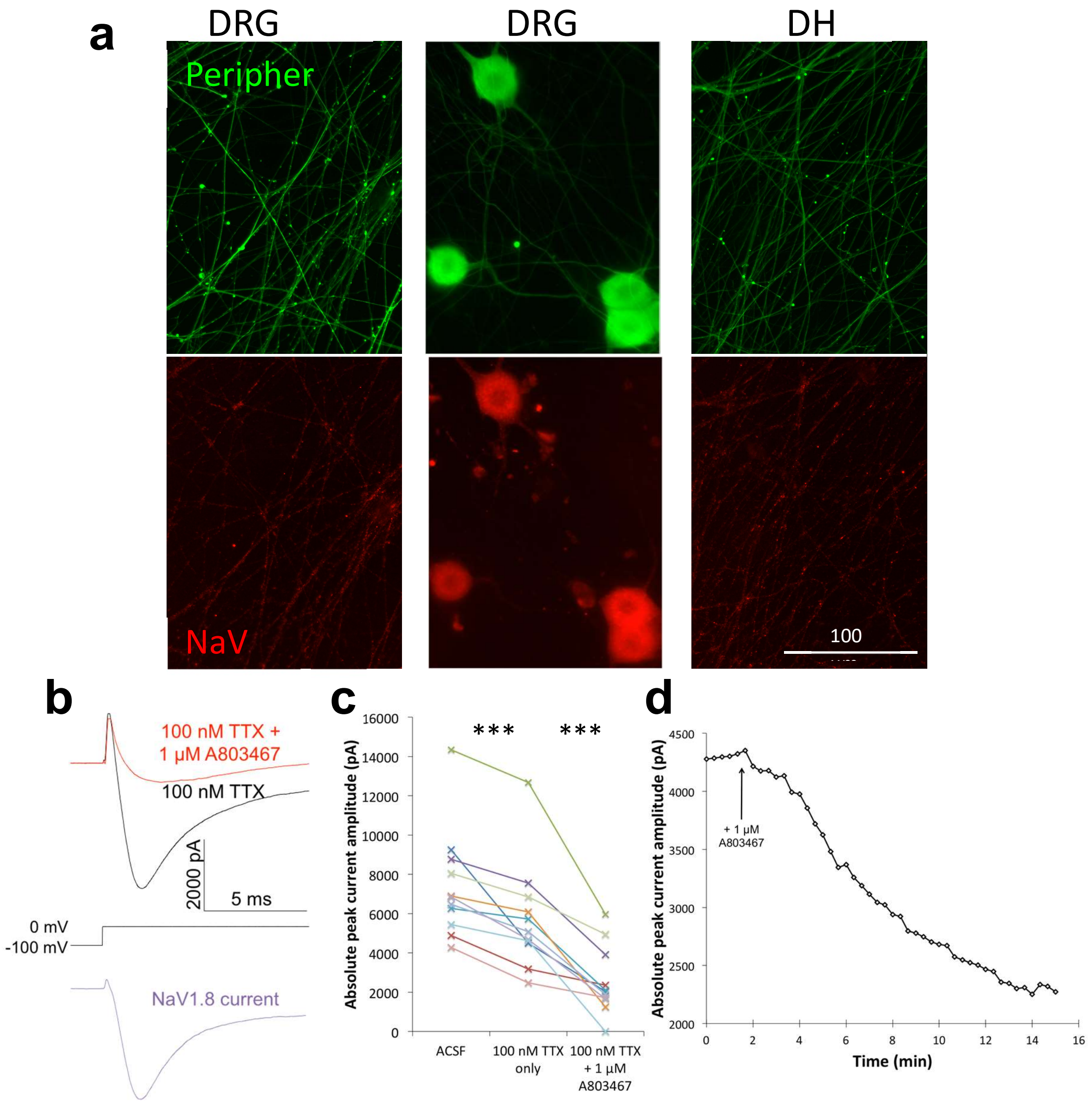

Figure S2. NaV1.8 is expressed in DRG soma and axons in the Periphery and DH compartments. (A) Rat DRGs from E16 embryos co-cultured with DH neurons for 12-16 DIV in microfluidic devices were stained for peripherin (green, top panels) and $Na_V1.8$ (red, bottom panels). The expression of $Na_V1.8$ in DRG cell bodies (bottom, middle panel) is highly variable between individual cells or cell types. Some of $Na_V1.8$ is delivered to axons in the Periphery (bottom left panel) and DH (bottom right panel) compartments. **(B)** The efficacy of $Na_V1.8$-specific blocker A803467 [1] was tested on rat E16 DRG neurons cultured for 11-13 DIV and analyzed for Na^+ currents by transient depolarization to 0 mV (see Methods for details). TTXr Na^+ currents (in presence of 100 nM TTX, black trace) are partially blocked by application 1 µM A803467 (red trace). Purple trace below shows the difference (subtraction) between the two curves. **(C)** Quantification of absolute peak current amplitudes reveals that not only TTX is effective at reducing some of the Na^+ currents in the DRGs, but that 1 µM A803467 partially blocks TTXr Na+ currents in some, but not all cells, supporting the variable degree of presence of $Na_V1.8$ in the DRG neurons, and showing the efficacy of the blockers used in **Figure 4**. (*** - $p < 0.001$, paired Student's *t*-test; $n = 11$ from 3 independent cultures). **(D)** Inhibition of TTXr Na+ currents (in presence of 500 nM TTX at the start of the trace) in cultured DRG neurons over time shows a significant reduction of Na^+ currents reaching a steady state within 12 minutes of A803467 application (representative recording, repeated $n = 3$).

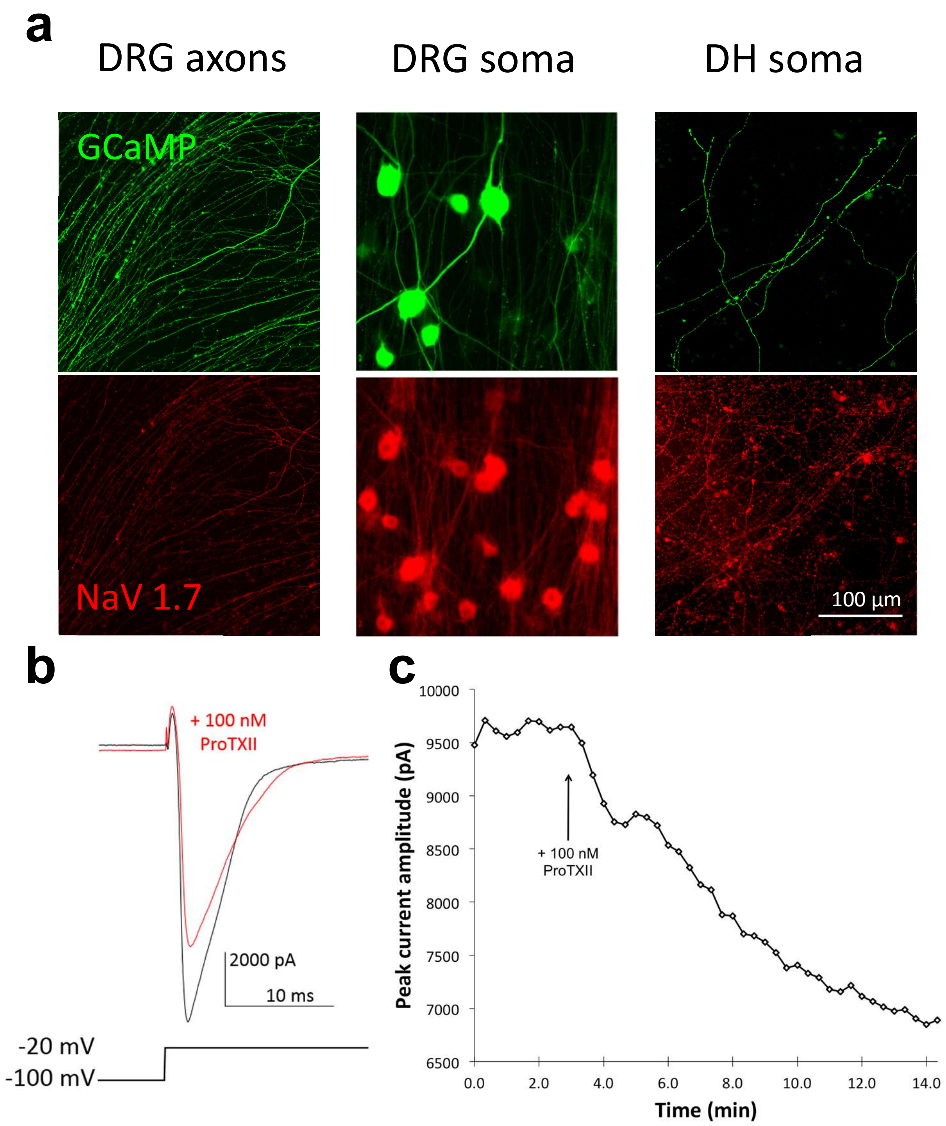

Figure S3. NaV1.7 is expressed on DRG neuron soma and axons in the Periphery and DH compartments. (A) DRG neurons from E16 rat embryos co-cultured with DH neurons in microfluidic devices for 12-16 DIV and infected with GCaMP at 6 DIV as described in Figure 6 were stained with anti-GFP antibody (recognizes GCaMP) in green, top panels and anti-Na_V1.7 in red, bottom panels. Representative images reveal that it was strongly expressed in all DRG cell bodies (middle panel) as well as evenly distributed throughout DRG axons in Periphery (left panel), and while also being evenly distributed along the axons from bilaterally crossing DRGs (GFP+ axons in top right panel) more punctate staining pattern was observed in the DH compartment. **(B)** The efficacy of ProTXII, a highly specific blocker of Na_V1.7 [2,3] was verified on DRG cells cultured on coverslips for 11-13 DIV and analyzed for Na^+ currents by transient depolarization to -20 mV (see Methods for details). The peak Na^+ current in presence of 1 µM A803467 (blocker of Na_V1.8, black trace) was reduced by application of 100 nM ProTXII in addition to A803467 (red trace, from representative experiment *n* = 3). **(C)** ProTXII-sensitive currents in cultured DRG neurons show a significant reduction, reaching a steady state within 10 minutes of ProTXII application (representative recording, repeated *n* = 3).

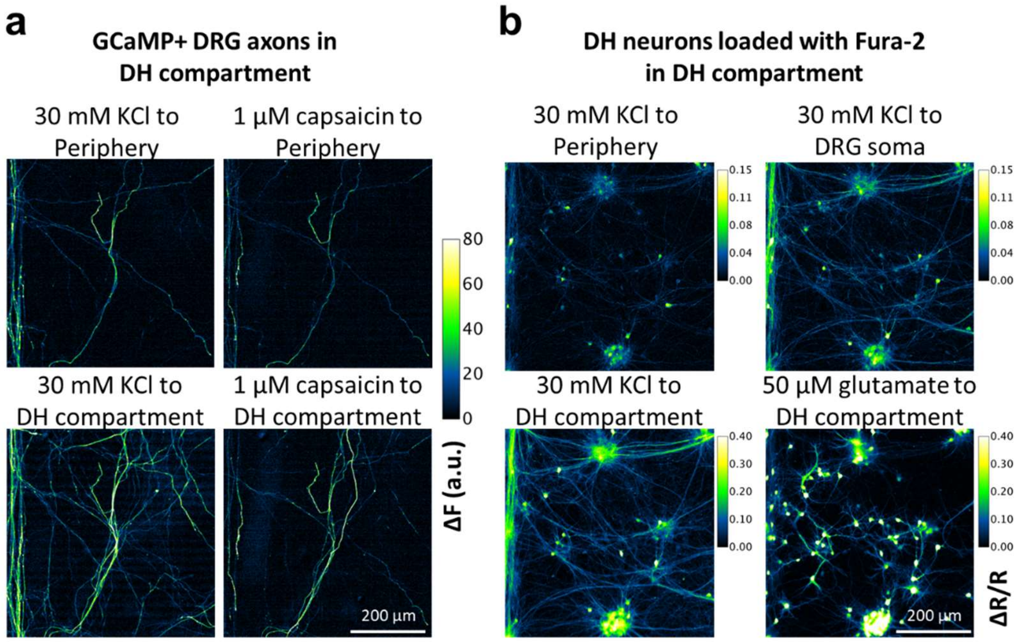

Figure S4. DRG axons in the DH compartment and DH neurons respond to stimulation of the Periphery compartment by KCl. (A) DRG axons expressing GCaMP (see Figure 6a for schematic) exhibit transient elevations of Ca^{2+} in response to depolarizing stimulus (30 mM KCl) applied to Periphery or directly (left panels, top and bottom respectively). Note, that a subset of these axons also responds to stimulation with TRPV1 agonist (1 μM capsaicin) applied to Periphery and directly (right panels, top and bottom respectively). **(B)** Cells in the DH compartment were loaded with ratiometric dye Fura-2 as shown in Figure 3. A chemical depolarizing stimulus applied to Periphery evokes Ca^{2+} transients in some DH neurons (top left panel) and chemically stimulating DRG cell bodies evokes a stronger response (top right panel). While direct application of 30 mM KCl to the imaged DH compartment stimulates DRG axons and some DH neurons (bottom left panel), direct application of 50 μM glutamate causes a strong response in DH neurons only (bottom right panel).

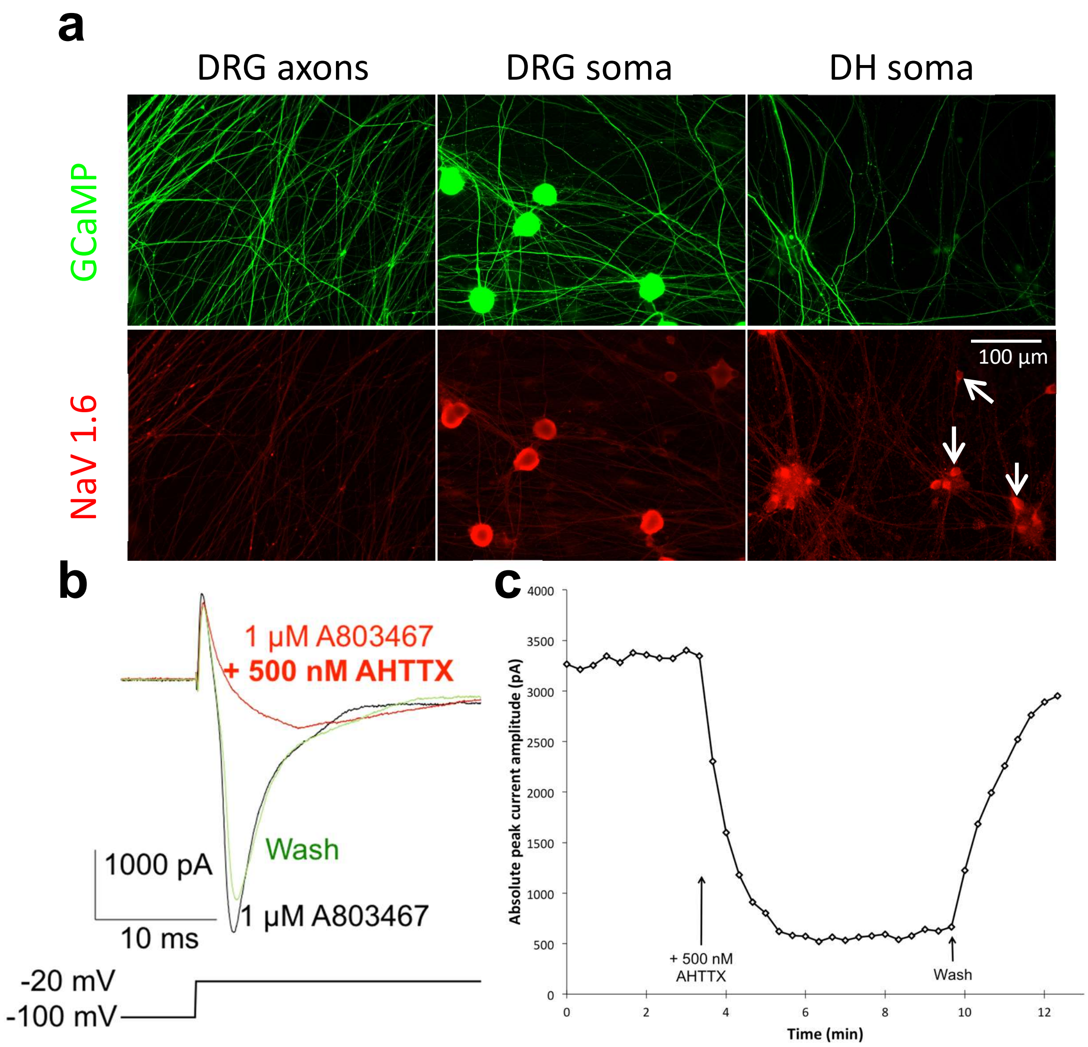

Figure S5. NaV1.6 is expressed by DRG neurons before and after axotomy. (A) Rat DRG neurons co-cultured with DH neurons in microfluidic devices for 11-12 DIV and labeled with AAV-GCaMP from Periphery at 6 DIV. The cells were stained with anti-GFP antibody (GCaMP, green) and anti-Na_V1.6 antibody (red). Note that there is significant expression of Na_V1.6 in DRG cell bodies in control as well as in DH cells (white arrows). **(B)** Na^+ currents in DRG cells cultured on coverslips for 11-13 DIV were recorded during delivery of -20 mV pulse in presence of 1 µM A803467 (to block Na_V1.8, black trace). Addition of 500 nM 4,9-anhydrotetrodotoxin (AHTTX, red trace) significantly reduces the Na^+ current and after wash (green trace) this current is almost completely restored (representative trace, *n* = 5 cells). **(C)** Inhibition of Na^+ current in a representative DRG cell over time after application of 500 nM AHTTX (1 µM A803467 is in bath solution throughout). Note that it reaches plateau within 2-3 minutes and washes out rapidly too.

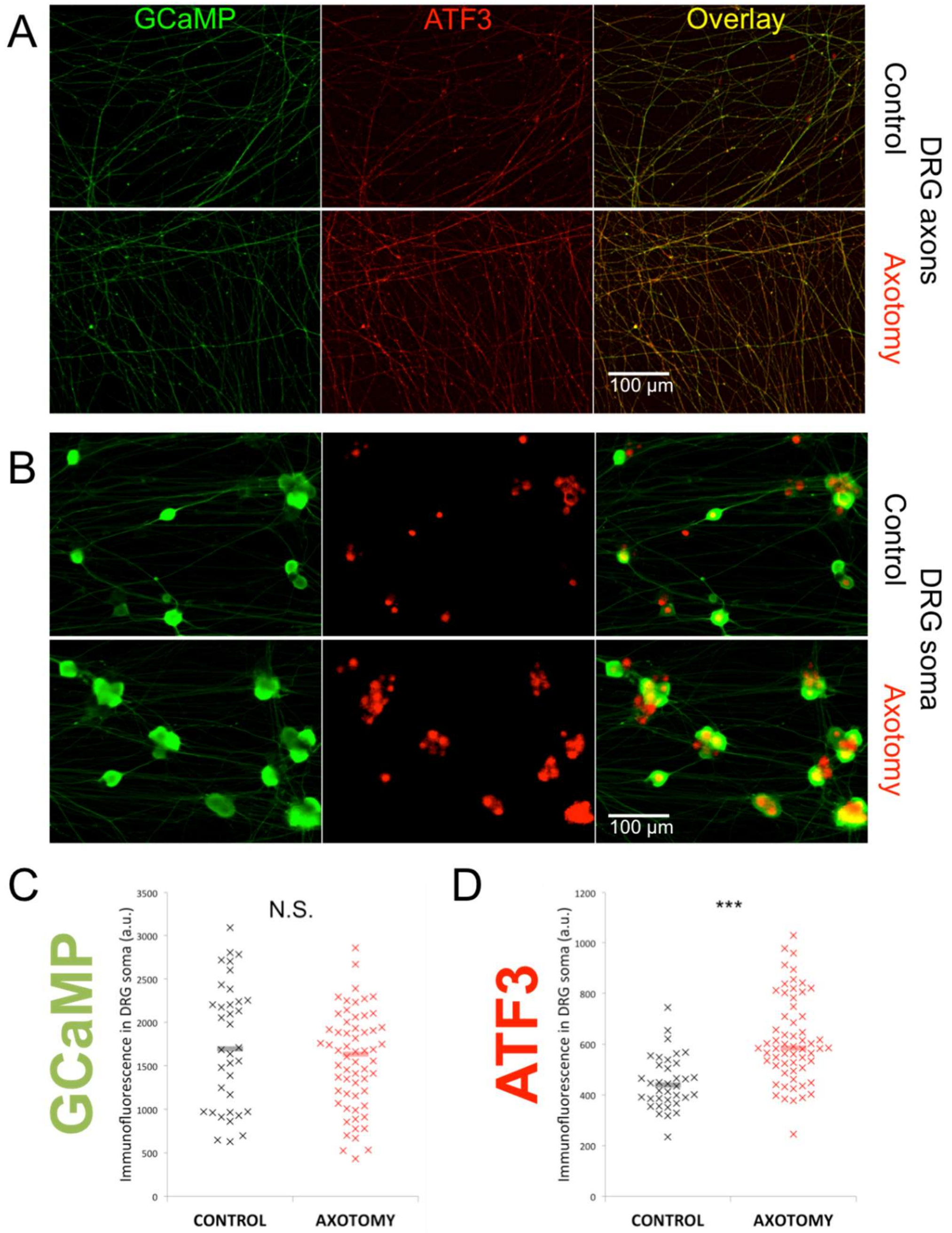

Figure S6. ATF3 is up-regulated in axotomised DRGs. Rat DRG neurons co-cultured with DH neurons in microfluidic devices for 11-12 DIV and labeled with AAV-GCaMP from Periphery at 6 DIV were axotomized (bottom panel, control on top panel) and allowed to recover from acute effects of axotomy for 3 DIV. The crossing neurons were then stained with the anti-GFP antibody (GCaMP, green) and anti-ATF3 antibody (red) as a marker of cell stress. **(A)** Representative images of axons in the Periphery (top panels) and DRG cell bodies (bottom panels) from cultures that have or have not been axotomized (*n* = 3 experiments). Note, that the axons, which have re-grown after axotomy, appear to have more ATF3 (red). Both cell bodies from axotomised cells and from control appear to express ATF3 in the nucleus, but there is also more ATF3 around the nucleus in the cytoplasm. **(D)** Quantification of ATF3 immunofluorescence in DRG soma shown from a representative experiment (*n* = 3 experiments) reveals a significant shift towards higher expression of ATF3 in axotomised neurons (red marks, *n* = 60 cells, red line represent the median) compared to those, which have crossed, but have not been axotomised (black marks, *n* = 36 cells, black line represents the median).

Note, that in the same culture, axotomy causes no change in immunofluorescence of our control protein (GCaMP; N.S., $p > 0.05$; ***, $p = 3.6 \times 10^{-8}$, Welch's *t*-test).

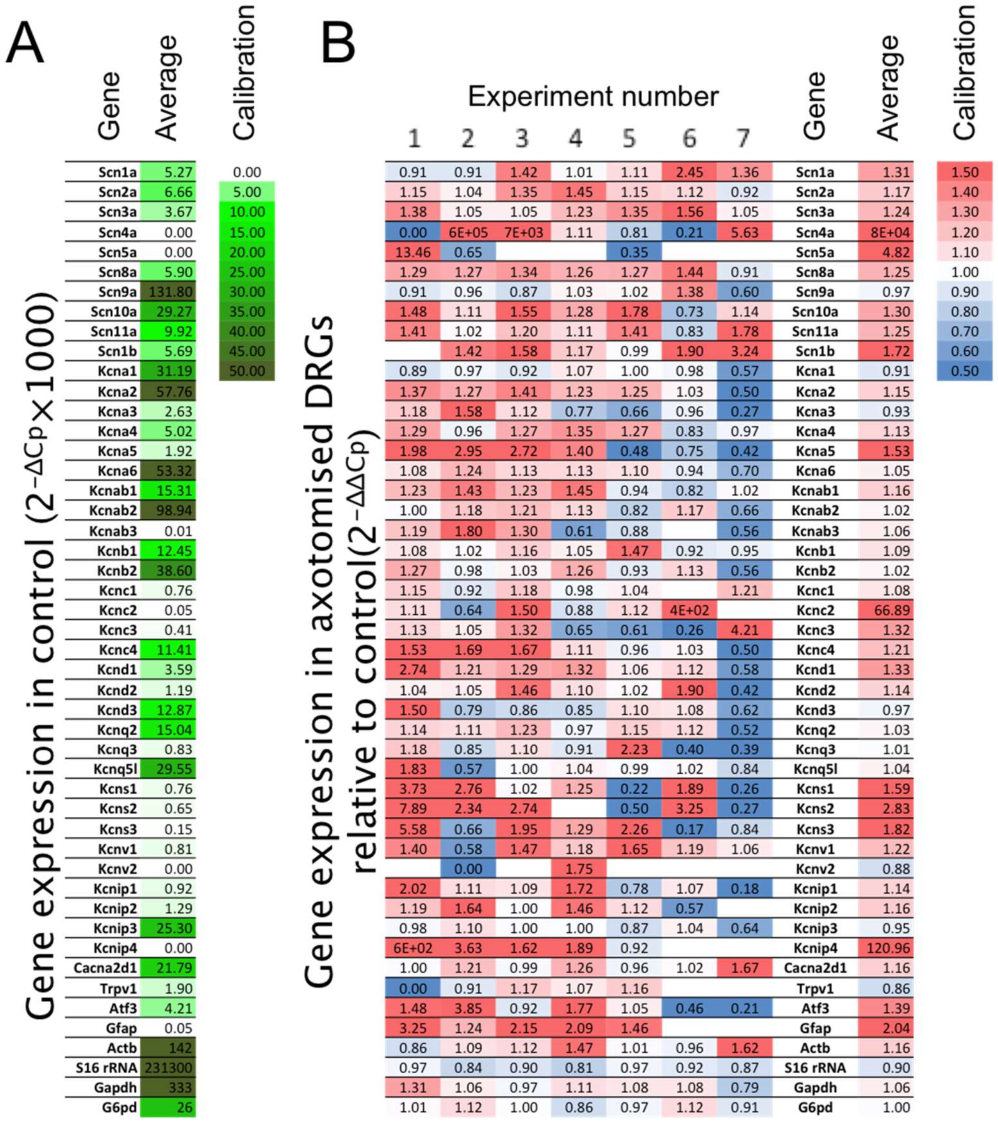

A Gene expression in control ($2^{-\Delta Cp} \times 1000$)

Gene	Average
Scn1a	5.27
Scn2a	6.66
Scn3a	3.67
Scn4a	0.00
Scn5a	0.00
Scn8a	5.90
Scn9a	131.80
Scn10a	29.27
Scn11a	9.92
Scn1b	5.69
Kcna1	31.19
Kcna2	57.76
Kcna3	2.63
Kcna4	5.02
Kcna5	1.92
Kcna6	53.32
Kcnab1	15.31
Kcnab2	98.94
Kcnab3	0.01
Kcnb1	12.45
Kcnb2	38.60
Kcnc1	0.76
Kcnc2	0.05
Kcnc3	0.41
Kcnc4	11.41
Kcnd1	3.59
Kcnd2	1.19
Kcnd3	12.87
Kcnq2	15.04
Kcnq3	0.83
Kcnq5l	29.55
Kcns1	0.76
Kcns2	0.65
Kcns3	0.15
Kcnv1	0.81
Kcnv2	0.00
Kcnip1	0.92
Kcnip2	1.29
Kcnip3	25.30
Kcnip4	0.00
Cacna2d1	21.79
Trpv1	1.90
Atf3	4.21
Gfap	0.05
Actb	142
S16 rRNA	231300
Gapdh	333
G6pd	26

Calibration
0.00
5.00
10.00
15.00
20.00
25.00
30.00
35.00
40.00
45.00
50.00

B Gene expression in axotomised DRGs relative to control($2^{-\Delta\Delta Cp}$)

Experiment number 1	2	3	4	5	6	7	Gene	Average
0.91	0.91	1.42	1.01	1.11	2.45	1.36	Scn1a	1.31
1.15	1.04	1.35	1.45	1.15	1.12	0.92	Scn2a	1.17
1.38	1.05	1.05	1.23	1.35	1.56	1.05	Scn3a	1.24
0.00	6E+05	7E+03	1.11	0.81	0.21	5.63	Scn4a	8E+04
13.46	0.65			0.35			Scn5a	4.82
1.29	1.27	1.34	1.26	1.27	1.44	0.91	Scn8a	1.25
0.91	0.96	0.87	1.03	1.02	1.38	0.60	Scn9a	0.97
1.48	1.11	1.55	1.28	1.78	0.73	1.14	Scn10a	1.30
1.41	1.02	1.20	1.11	1.41	0.83	1.78	Scn11a	1.25
	1.42	1.58	1.17	0.99	1.90	3.24	Scn1b	1.72
0.89	0.97	0.92	1.07	1.00	0.98	0.57	Kcna1	0.91
1.37	1.27	1.41	1.23	1.25	1.03	0.50	Kcna2	1.15
1.18	1.58	1.12	0.77	0.66	0.96	0.27	Kcna3	0.93
1.29	0.96	1.27	1.35	1.27	0.83	0.97	Kcna4	1.13
1.98	2.95	2.72	1.40	0.48	0.75	0.42	Kcna5	1.53
1.08	1.24	1.13	1.13	1.10	0.94	0.70	Kcna6	1.05
1.23	1.43	1.23	1.45	0.94	0.82	1.02	Kcnab1	1.16
1.00	1.18	1.21	1.13	0.82	1.17	0.66	Kcnab2	1.02
1.19	1.80	1.30	0.61	0.88		0.56	Kcnab3	1.06
1.08	1.02	1.16	1.05	1.47	0.92	0.95	Kcnb1	1.09
1.27	0.98	1.03	1.26	0.93	1.13	0.56	Kcnb2	1.02
1.15	0.92	1.18	0.98	1.04		1.21	Kcnc1	1.08
1.11	0.64	1.50	0.88	1.12	4E+02		Kcnc2	66.89
1.13	1.05	1.32	0.65	0.61	0.26	4.21	Kcnc3	1.32
1.53	1.69	1.67	1.11	0.96	1.03	0.50	Kcnc4	1.21
2.74	1.21	1.29	1.32	1.06	1.12	0.58	Kcnd1	1.33
1.04	1.05	1.46	1.10	1.02	1.90	0.42	Kcnd2	1.14
1.50	0.79	0.86	0.85	1.10	1.08	0.62	Kcnd3	0.97
1.14	1.11	1.23	0.97	1.15	1.12	0.52	Kcnq2	1.03
1.18	0.85	1.10	0.91	2.23	0.40	0.39	Kcnq3	1.01
1.83	0.57	1.00	1.04	0.99	1.02	0.84	Kcnq5l	1.04
3.73	2.76	1.02	1.25	0.22	1.89	0.26	Kcns1	1.59
7.89	2.34	2.74		0.50	3.25	0.27	Kcns2	2.83
5.58	0.66	1.95	1.29	2.26	0.17	0.84	Kcns3	1.82
1.40	0.58	1.47	1.18	1.65	1.19	1.06	Kcnv1	1.22
	0.00		1.75				Kcnv2	0.88
2.02	1.11	1.09	1.72	0.78	1.07	0.18	Kcnip1	1.14
1.19	1.64	1.00	1.46	1.12	0.57		Kcnip2	1.16
0.98	1.10	1.00	1.00	0.87	1.04	0.64	Kcnip3	0.95
6E+02	3.63	1.62	1.89	0.92			Kcnip4	120.96
1.00	1.21	0.99	1.26	0.96	1.02	1.67	Cacna2d1	1.16
0.00	0.91	1.17	1.07	1.16			Trpv1	0.86
1.48	3.85	0.92	1.77	1.05	0.46	0.21	Atf3	1.39
3.25	1.24	2.15	2.09	1.46			Gfap	2.04
0.86	1.09	1.12	1.47	1.01	0.96	1.62	Actb	1.16
0.97	0.84	0.90	0.81	0.97	0.92	0.87	S16 rRNA	0.90
1.31	1.06	0.97	1.11	1.08	1.08	0.79	Gapdh	1.06
1.01	1.12	1.00	0.86	0.97	1.12	0.91	G6pd	1.00

Calibration
1.50
1.40
1.30
1.20
1.10
1.00
0.90
0.80
0.70
0.60
0.50

Figure S7. Gene expression quantification in axotomized neurons. DRG and DH neurons were co-cultured for 12-13 DIV, axotomized and allowed to recover in the incubator for 3 DIV. Then, the DRGs were harvested for qPCR analysis as shown in Figure 5. Geometric mean of *Actb*, *Gapdh*, *G6pd*, and 16S rRNA was used as reference for normalizing expression of all the genes. **(A)** The crossing points (Cp) in control cultures were converted to relative gene expression levels on a linear scale by $2^{-\Delta Cp}$ method and multiplied by 1000 for representation. Genes in dark green are highly expressed, while genes in light green are expressed at low levels. Note that *Scn9a* is the most highly expressed Na_V in our DRGs, while other Na_Vs (except *Scn4a* and *Scn5a*) are moderately expressed. **(B)** Changes in expression of the genes in axotomized cultures relative to control ($2^{-\Delta\Delta Cp}$ method) for each experiment performed (*n* = 7), and average (column to the right). Up-regulated genes are shown in red, while down-regulated genes are in blue (empty cells denote where Cp could not be determined).

Supplementary References

1 Jarvis, M. F. *et al.* A-803467, a potent and selective Nav1.8 sodium channel blocker, attenuates neuropathic and inflammatory pain in the rat. *Proc Natl Acad Sci U S A* **104**, 8520-8525 (2007).

2 Schmalhofer, W. A. *et al.* ProTx-II, a selective inhibitor of NaV1.7 sodium channels, blocks action potential propagation in nociceptors. *Mol Pharmacol* **74**, 1476-1484 (2008).

3 Flinspach, M. *et al.* Insensitivity to pain induced by a potent selective closed-state Nav1.7 inhibitor. *Sci Rep* **7**, 39662, https://doi.org/10.1038/srep39662 (2017).
